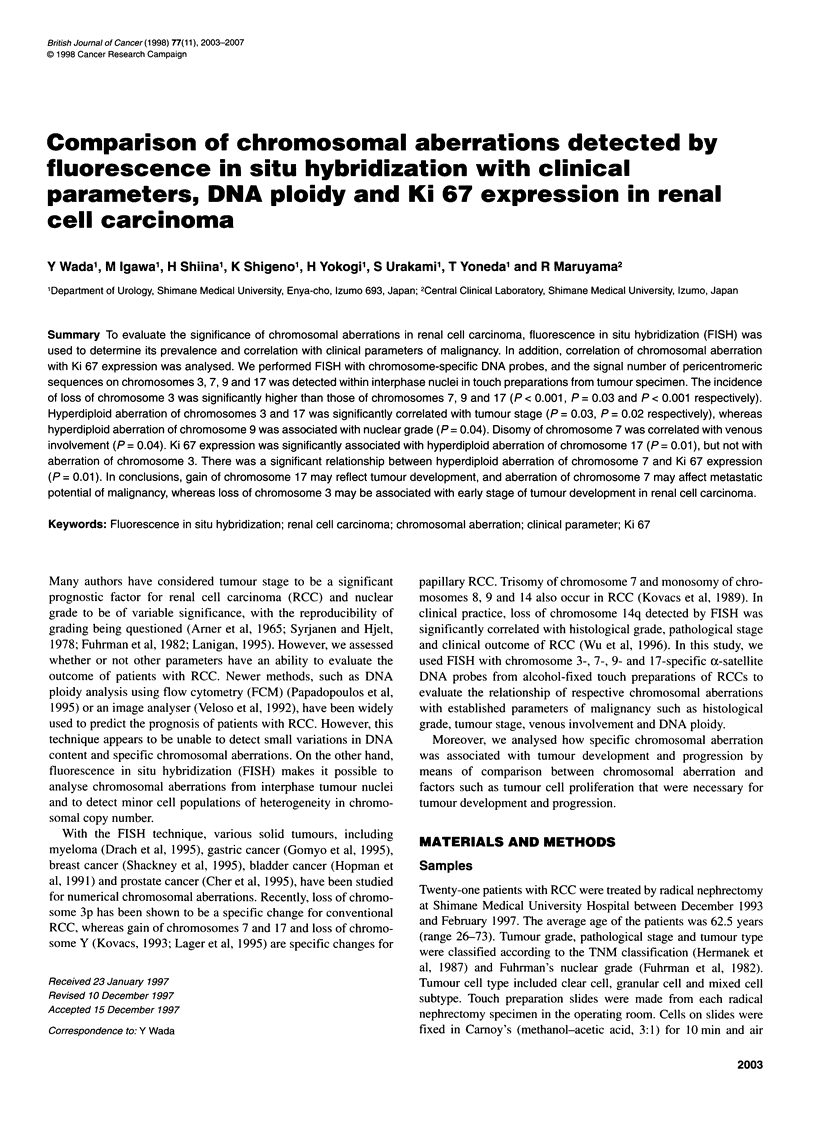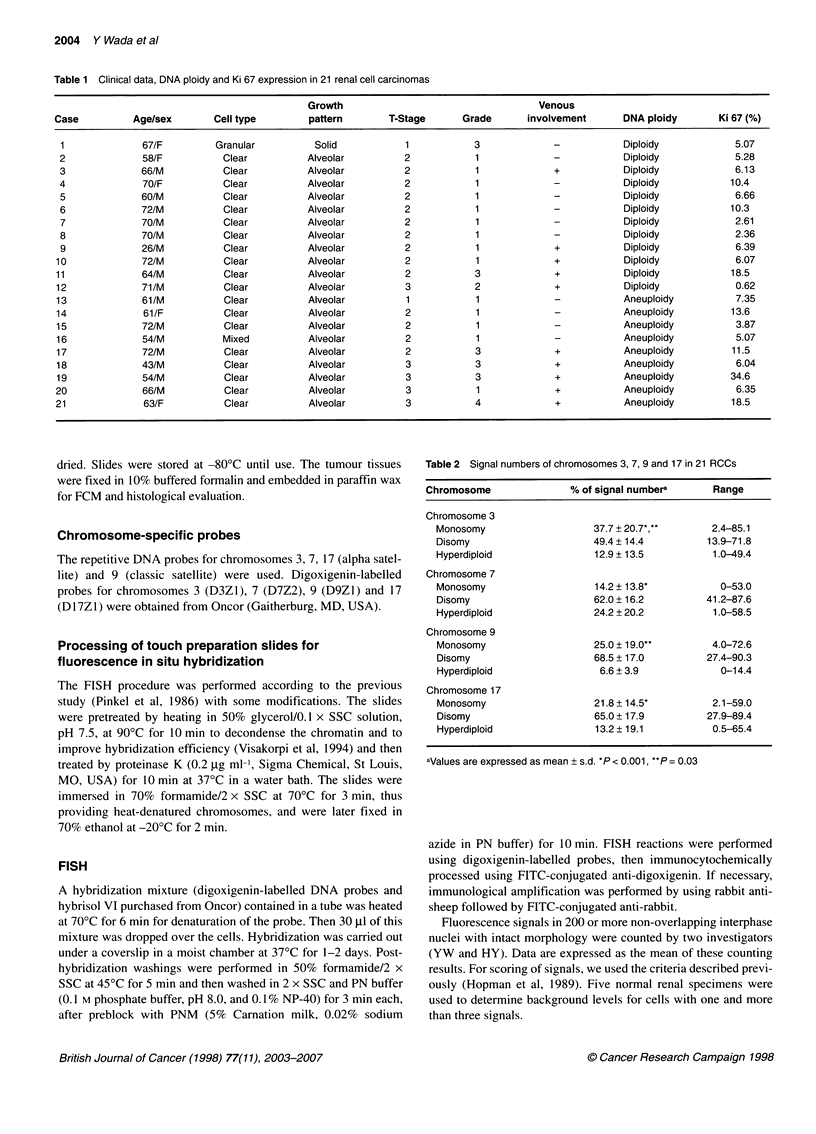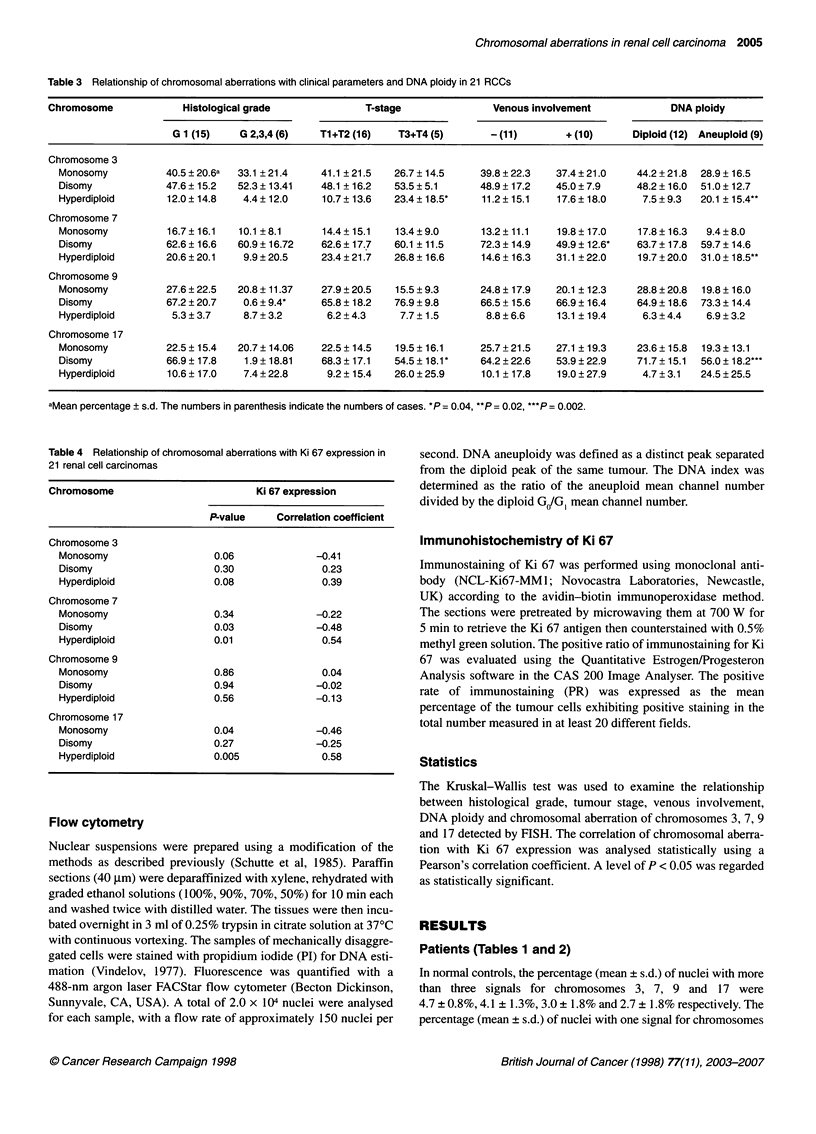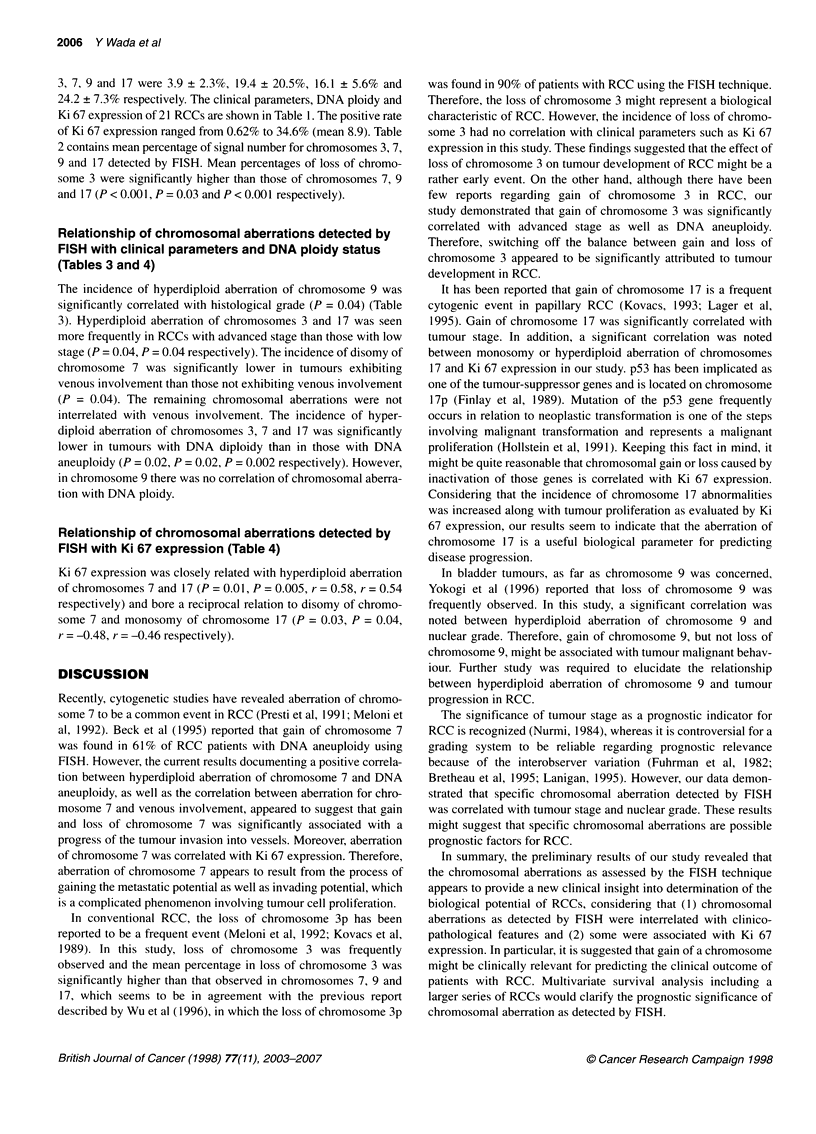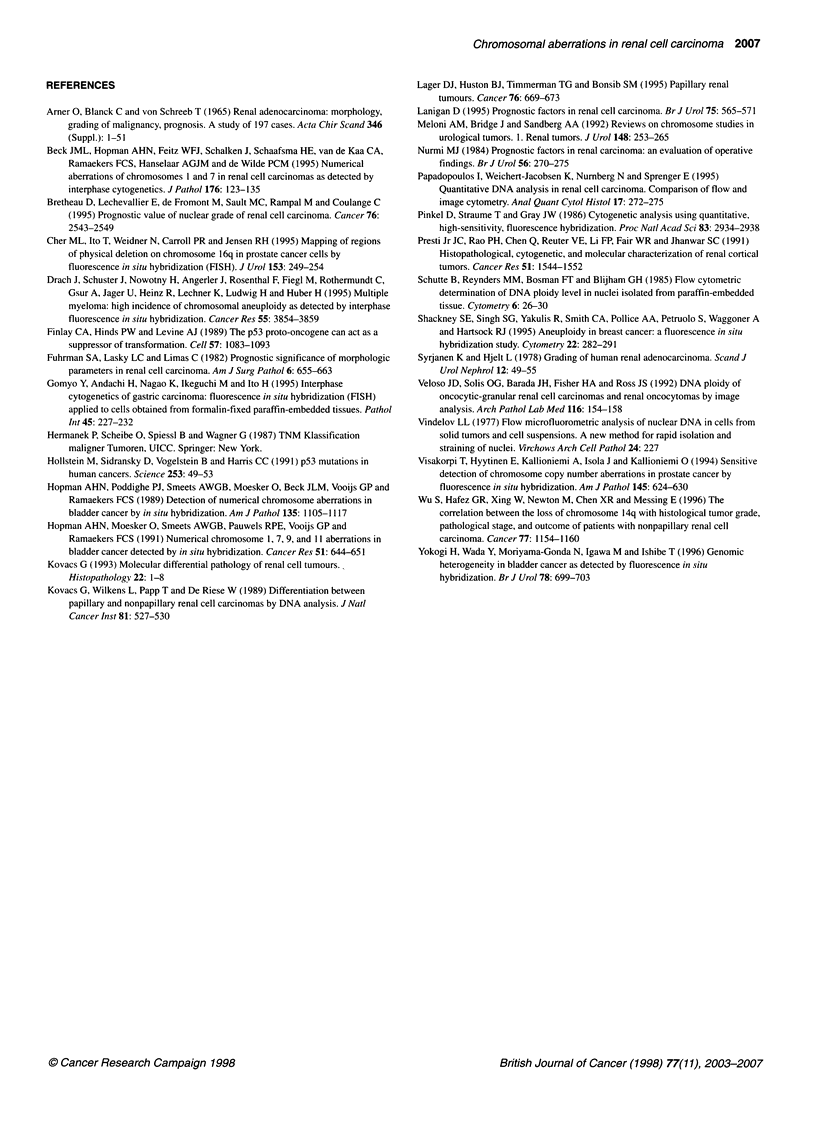# Comparison of chromosomal aberrations detected by fluorescence in situ hybridization with clinical parameters, DNA ploidy and Ki 67 expression in renal cell carcinoma.

**DOI:** 10.1038/bjc.1998.332

**Published:** 1998-06

**Authors:** Y. Wada, M. Igawa, H. Shiina, K. Shigeno, H. Yokogi, S. Urakami, T. Yoneda, R. Maruyama

**Affiliations:** Department of Urology, Shimane Medical University, Izumo, Japan.

## Abstract

To evaluate the significance of chromosomal aberrations in renal cell carcinoma, fluorescence in situ hybridization (FISH) was used to determine its prevalence and correlation with clinical parameters of malignancy. In addition, correlation of chromosomal aberration with Ki 67 expression was analysed. We performed FISH with chromosome-specific DNA probes, and the signal number of pericentromeric sequences on chromosomes 3, 7, 9 and 17 was detected within interphase nuclei in touch preparations from tumour specimen. The incidence of loss of chromosome 3 was significantly higher than those of chromosomes 7, 9 and 17 (P < 0.001, P = 0.03 and P < 0.001 respectively). Hyperdiploid aberration of chromosomes 3 and 17 was significantly correlated with tumour stage (P = 0.03, P = 0.02 respectively), whereas hyperdiploid aberration of chromosome 9 was associated with nuclear grade (P = 0.04). Disomy of chromosome 7 was correlated with venous involvement (P = 0.04). Ki 67 expression was significantly associated with hyperdiploid aberration of chromosome 17 (P = 0.01), but not with aberration of chromosome 3. There was a significant relationship between hyperdiploid aberration of chromosome 7 and Ki 67 expression (P = 0.01). In conclusions, gain of chromosome 17 may reflect tumour development, and aberration of chromosome 7 may affect metastatic potential of malignancy, whereas loss of chromosome 3 may be associated with early stage of tumour development in renal cell carcinoma.


British Journal of Cancer (1998) 77(11), 2003–2007
© 1998 Cancer Research Campaign

### Comparison of chromosomal aberrations detected by fluorescence in situ hybridization with clinical parameters, DNA ploidy and Ki 67 expression in renal cell carcinoma

**Y Wada[1], M Igawa[1], H Shiina[1], K Shigeno[1], H Yokogi[1], S Urakami[1], T Yoneda[1] and R Maruyama[2]**

[1]Department of Urology, Shimane Medical University, Enya-cho, Izumo 693, Japan; [2]Central Clinical Laboratory, Shimane Medical University, Izumo, Japan

**Summary** To evaluate the significance of chromosomal aberrations in renal cell carcinoma, fluorescence in situ hybridization (FISH) was used to determine its prevalence and correlation with clinical parameters of malignancy. In addition, correlation of chromosomal aberration with Ki 67 expression was analysed. We performed FISH with chromosome-specific DNA probes, and the signal number of pericentromeric sequences on chromosomes 3, 7, 9 and 17 was detected within interphase nuclei in touch preparations from tumour specimen. The incidence of loss of chromosome 3 was significantly higher than those of chromosomes 7, 9 and 17 ($P < 0.001$, $P = 0.03$ and $P < 0.001$ respectively). Hyperdiploid aberration of chromosomes 3 and 17 was significantly correlated with tumour stage ($P = 0.03$, $P = 0.02$ respectively), whereas hyperdiploid aberration of chromosome 9 was associated with nuclear grade ($P = 0.04$). Disomy of chromosome 7 was correlated with venous involvement ($P = 0.04$). Ki 67 expression was significantly associated with hyperdiploid aberration of chromosome 17 ($P = 0.01$), but not with aberration of chromosome 3. There was a significant relationship between hyperdiploid aberration of chromosome 7 and Ki 67 expression ($P = 0.01$). In conclusions, gain of chromosome 17 may reflect tumour development, and aberration of chromosome 7 may affect metastatic potential of malignancy, whereas loss of chromosome 3 may be associated with early stage of tumour development in renal cell carcinoma.

**Keywords:** Fluorescence in situ hybridization; renal cell carcinoma; chromosomal aberration; clinical parameter; Ki 67

Many authors have considered tumour stage to be a significant prognostic factor for renal cell carcinoma (RCC) and nuclear grade to be of variable significance, with the reproducibility of grading being questioned (Arner et al, 1965; Syrjanen and Hjelt, 1978; Fuhrman et al, 1982; Lanigan, 1995). However, we assessed whether or not other parameters have an ability to evaluate the outcome of patients with RCC. Newer methods, such as DNA ploidy analysis using flow cytometry (FCM) (Papadopoulos et al, 1995) or an image analyser (Veloso et al, 1992), have been widely used to predict the prognosis of patients with RCC. However, this technique appears to be unable to detect small variations in DNA content and specific chromosomal aberrations. On the other hand, fluorescence in situ hybridization (FISH) makes it possible to analyse chromosomal aberrations from interphase tumour nuclei and to detect minor cell populations of heterogeneity in chromosomal copy number.

With the FISH technique, various solid tumours, including myeloma (Drach et al, 1995), gastric cancer (Gomyo et al, 1995), breast cancer (Shackney et al, 1995), bladder cancer (Hopman et al, 1991) and prostate cancer (Cher et al, 1995), have been studied for numerical chromosomal aberrations. Recently, loss of chromosome 3p has been shown to be a specific change for conventional RCC, whereas gain of chromosomes 7 and 17 and loss of chromosome Y (Kovacs, 1993; Lager et al, 1995) are specific changes for papillary RCC. Trisomy of chromosome 7 and monosomy of chromosomes 8, 9 and 14 also occur in RCC (Kovacs et al, 1989). In clinical practice, loss of chromosome 14q detected by FISH was significantly correlated with histological grade, pathological stage and clinical outcome of RCC (Wu et al, 1996). In this study, we used FISH with chromosome 3-, 7-, 9- and 17-specific α-satellite DNA probes from alcohol-fixed touch preparations of RCCs to evaluate the relationship of respective chromosomal aberrations with established parameters of malignancy such as histological grade, tumour stage, venous involvement and DNA ploidy.

Moreover, we analysed how specific chromosomal aberration was associated with tumour development and progression by means of comparison between chromosomal aberration and factors such as tumour cell proliferation that were necessary for tumour development and progression.

*Received 23 January 1997*
*Revised 10 December 1997*
*Accepted 15 December 1997*

*Correspondence to:* Y Wada

### MATERIALS AND METHODS

### Samples

Twenty-one patients with RCC were treated by radical nephrectomy at Shimane Medical University Hospital between December 1993 and February 1997. The average age of the patients was 62.5 years (range 26–73). Tumour grade, pathological stage and tumour type were classified according to the TNM classification (Hermanek et al, 1987) and Fuhrman's nuclear grade (Fuhrman et al, 1982). Tumour cell type included clear cell, granular cell and mixed cell subtype. Touch preparation slides were made from each radical nephrectomy specimen in the operating room. Cells on slides were fixed in Carnoy's (methanol–acetic acid, 3:1) for 10 min and air

Table 1 Clinical data, DNA ploidy and Ki 67 expression in 21 renal cell carcinomas

| Case | Age/sex | Cell type | Growth pattern | T-Stage | Grade | Venous involvement | DNA ploidy | Ki 67 (%) |
|---|---|---|---|---|---|---|---|---|
| 1 | 67/F | Granular | Solid | 1 | 3 | – | Diploidy | 5.07 |
| 2 | 58/F | Clear | Alveolar | 2 | 1 | – | Diploidy | 5.28 |
| 3 | 66/M | Clear | Alveolar | 2 | 1 | + | Diploidy | 6.13 |
| 4 | 70/F | Clear | Alveolar | 2 | 1 | – | Diploidy | 10.4 |
| 5 | 60/M | Clear | Alveolar | 2 | 1 | – | Diploidy | 6.66 |
| 6 | 72/M | Clear | Alveolar | 2 | 1 | – | Diploidy | 10.3 |
| 7 | 70/M | Clear | Alveolar | 2 | 1 | – | Diploidy | 2.61 |
| 8 | 70/M | Clear | Alveolar | 2 | 1 | – | Diploidy | 2.36 |
| 9 | 26/M | Clear | Alveolar | 2 | 1 | + | Diploidy | 6.39 |
| 10 | 72/M | Clear | Alveolar | 2 | 1 | + | Diploidy | 6.07 |
| 11 | 64/M | Clear | Alveolar | 2 | 3 | + | Diploidy | 18.5 |
| 12 | 71/M | Clear | Alveolar | 3 | 2 | + | Diploidy | 0.62 |
| 13 | 61/M | Clear | Alveolar | 1 | 1 | – | Aneuploidy | 7.35 |
| 14 | 61/F | Clear | Alveolar | 2 | 1 | – | Aneuploidy | 13.6 |
| 15 | 72/M | Clear | Alveolar | 2 | 1 | – | Aneuploidy | 3.87 |
| 16 | 54/M | Mixed | Alveolar | 2 | 1 | – | Aneuploidy | 5.07 |
| 17 | 72/M | Clear | Alveolar | 2 | 3 | + | Aneuploidy | 11.5 |
| 18 | 43/M | Clear | Alveolar | 3 | 3 | + | Aneuploidy | 6.04 |
| 19 | 54/M | Clear | Alveolar | 3 | 3 | + | Aneuploidy | 34.6 |
| 20 | 66/M | Clear | Alveolar | 3 | 1 | + | Aneuploidy | 6.35 |
| 21 | 63/F | Clear | Alveolar | 3 | 4 | + | Aneuploidy | 18.5 |

dried. Slides were stored at –80°C until use. The tumour tissues were fixed in 10% buffered formalin and embedded in paraffin wax for FCM and histological evaluation.

### Chromosome-specific probes

The repetitive DNA probes for chromosomes 3, 7, 17 (alpha satellite) and 9 (classic satellite) were used. Digoxigenin-labelled probes for chromosomes 3 (D3Z1), 7 (D7Z2), 9 (D9Z1) and 17 (D17Z1) were obtained from Oncor (Gaitherburg, MD, USA).

### Processing of touch preparation slides for fluorescence in situ hybridization

The FISH procedure was performed according to the previous study (Pinkel et al, 1986) with some modifications. The slides were pretreated by heating in 50% glycerol/0.1 × SSC solution, pH 7.5, at 90°C for 10 min to decondense the chromatin and to improve hybridization efficiency (Visakorpi et al, 1994) and then treated by proteinase K (0.2 μg ml$^{-1}$, Sigma Chemical, St Louis, MO, USA) for 10 min at 37°C in a water bath. The slides were immersed in 70% formamide/2 × SSC at 70°C for 3 min, thus providing heat-denatured chromosomes, and were later fixed in 70% ethanol at –20°C for 2 min.

### FISH

A hybridization mixture (digoxigenin-labelled DNA probes and hybrisol VI purchased from Oncor) contained in a tube was heated at 70°C for 6 min for denaturation of the probe. Then 30 μl of this mixture was dropped over the cells. Hybridization was carried out under a coverslip in a moist chamber at 37°C for 1–2 days. Post-hybridization washings were performed in 50% formamide/2 × SSC at 45°C for 5 min and then washed in 2 × SSC and PN buffer (0.1 M phosphate buffer, pH 8.0, and 0.1% NP-40) for 3 min each, after preblock with PNM (5% Carnation milk, 0.02% sodium azide in PN buffer) for 10 min. FISH reactions were performed using digoxigenin-labelled probes, then immunocytochemically processed using FITC-conjugated anti-digoxigenin. If necessary, immunological amplification was performed by using rabbit anti-sheep followed by FITC-conjugated anti-rabbit.

Fluorescence signals in 200 or more non-overlapping interphase nuclei with intact morphology were counted by two investigators (YW and HY). Data are expressed as the mean of these counting results. For scoring of signals, we used the criteria described previously (Hopman et al, 1989). Five normal renal specimens were used to determine background levels for cells with one and more than three signals.

Table 2 Signal numbers of chromosomes 3, 7, 9 and 17 in 21 RCCs

| Chromosome | % of signal number[a] | Range |
|---|---|---|
| Chromosome 3 | | |
| Monosomy | 37.7 ± 20.7*,** | 2.4–85.1 |
| Disomy | 49.4 ± 14.4 | 13.9–71.8 |
| Hyperdiploid | 12.9 ± 13.5 | 1.0–49.4 |
| Chromosome 7 | | |
| Monosomy | 14.2 ± 13.8* | 0–53.0 |
| Disomy | 62.0 ± 16.2 | 41.2–87.6 |
| Hyperdiploid | 24.2 ± 20.2 | 1.0–58.5 |
| Chromosome 9 | | |
| Monosomy | 25.0 ± 19.0** | 4.0–72.6 |
| Disomy | 68.5 ± 17.0 | 27.4–90.3 |
| Hyperdiploid | 6.6 ± 3.9 | 0–14.4 |
| Chromosome 17 | | |
| Monosomy | 21.8 ± 14.5* | 2.1–59.0 |
| Disomy | 65.0 ± 17.9 | 27.9–89.4 |
| Hyperdiploid | 13.2 ± 19.1 | 0.5–65.4 |

[a]Values are expressed as mean ± s.d. *$P < 0.001$, **$P = 0.03$

© Cancer Research Campaign 1998

**Table 3** Relationship of chromosomal aberrations with clinical parameters and DNA ploidy in 21 RCCs

| Chromosome | Histological grade | | T-stage | | Venous involvement | | DNA ploidy | |
|---|---|---|---|---|---|---|---|---|
| | G 1 (15) | G 2,3,4 (6) | T1+T2 (16) | T3+T4 (5) | – (11) | + (10) | Diploid (12) | Aneuploid (9) |
| Chromosome 3 | | | | | | | | |
| Monosomy | 40.5 ± 20.6[a] | 33.1 ± 21.4 | 41.1 ± 21.5 | 26.7 ± 14.5 | 39.8 ± 22.3 | 37.4 ± 21.0 | 44.2 ± 21.8 | 28.9 ± 16.5 |
| Disomy | 47.6 ± 15.2 | 52.3 ± 13.41 | 48.1 ± 16.2 | 53.5 ± 5.1 | 48.9 ± 17.2 | 45.0 ± 7.9 | 48.2 ± 16.0 | 51.0 ± 12.7 |
| Hyperdiploid | 12.0 ± 14.8 | 4.4 ± 12.0 | 10.7 ± 13.6 | 23.4 ± 18.5* | 11.2 ± 15.1 | 17.6 ± 18.0 | 7.5 ± 9.3 | 20.1 ± 15.4** |
| Chromosome 7 | | | | | | | | |
| Monosomy | 16.7 ± 16.1 | 10.1 ± 8.1 | 14.4 ± 15.1 | 13.4 ± 9.0 | 13.2 ± 11.1 | 19.8 ± 17.0 | 17.8 ± 16.3 | 9.4 ± 8.0 |
| Disomy | 62.6 ± 16.6 | 60.9 ± 16.72 | 62.6 ± 17.7 | 60.1 ± 11.5 | 72.3 ± 14.9 | 49.9 ± 12.6* | 63.7 ± 17.8 | 59.7 ± 14.6 |
| Hyperdiploid | 20.6 ± 20.1 | 9.9 ± 20.5 | 23.4 ± 21.7 | 26.8 ± 16.6 | 14.6 ± 16.3 | 31.1 ± 22.0 | 19.7 ± 20.0 | 31.0 ± 18.5** |
| Chromosome 9 | | | | | | | | |
| Monosomy | 27.6 ± 22.5 | 20.8 ± 11.37 | 27.9 ± 20.5 | 15.5 ± 9.3 | 24.8 ± 17.9 | 20.1 ± 12.3 | 28.8 ± 20.8 | 19.8 ± 16.0 |
| Disomy | 67.2 ± 20.7 | 0.6 ± 9.4* | 65.8 ± 18.2 | 76.9 ± 9.8 | 66.5 ± 15.6 | 66.9 ± 16.4 | 64.9 ± 18.6 | 73.3 ± 14.4 |
| Hyperdiploid | 5.3 ± 3.7 | 8.7 ± 3.2 | 6.2 ± 4.3 | 7.7 ± 1.5 | 8.8 ± 6.6 | 13.1 ± 19.4 | 6.3 ± 4.4 | 6.9 ± 3.2 |
| Chromosome 17 | | | | | | | | |
| Monosomy | 22.5 ± 15.4 | 20.7 ± 14.06 | 22.5 ± 14.5 | 19.5 ± 16.1 | 25.7 ± 21.5 | 27.1 ± 19.3 | 23.6 ± 15.8 | 19.3 ± 13.1 |
| Disomy | 66.9 ± 17.8 | 1.9 ± 18.81 | 68.3 ± 17.1 | 54.5 ± 18.1* | 64.2 ± 22.6 | 53.9 ± 22.9 | 71.7 ± 15.1 | 56.0 ± 18.2*** |
| Hyperdiploid | 10.6 ± 17.0 | 7.4 ± 22.8 | 9.2 ± 15.4 | 26.0 ± 25.9 | 10.1 ± 17.8 | 19.0 ± 27.9 | 4.7 ± 3.1 | 24.5 ± 25.5 |

[a]Mean percentage ± s.d. The numbers in parenthesis indicate the numbers of cases. *$P = 0.04$, **$P = 0.02$, ***$P = 0.002$.

**Table 4** Relationship of chromosomal aberrations with Ki 67 expression in 21 renal cell carcinomas

| Chromosome | Ki 67 expression | |
|---|---|---|
| | *P*-value | Correlation coefficient |
| Chromosome 3 | | |
| Monosomy | 0.06 | –0.41 |
| Disomy | 0.30 | 0.23 |
| Hyperdiploid | 0.08 | 0.39 |
| Chromosome 7 | | |
| Monosomy | 0.34 | –0.22 |
| Disomy | 0.03 | –0.48 |
| Hyperdiploid | 0.01 | 0.54 |
| Chromosome 9 | | |
| Monosomy | 0.86 | 0.04 |
| Disomy | 0.94 | –0.02 |
| Hyperdiploid | 0.56 | –0.13 |
| Chromosome 17 | | |
| Monosomy | 0.04 | –0.46 |
| Disomy | 0.27 | –0.25 |
| Hyperdiploid | 0.005 | 0.58 |

### Flow cytometry

Nuclear suspensions were prepared using a modification of the methods as described previously (Schutte et al, 1985). Paraffin sections (40 μm) were deparaffinized with xylene, rehydrated with graded ethanol solutions (100%, 90%, 70%, 50%) for 10 min each and washed twice with distilled water. The tissues were then incubated overnight in 3 ml of 0.25% trypsin in citrate solution at 37°C with continuous vortexing. The samples of mechanically disaggregated cells were stained with propidium iodide (PI) for DNA estimation (Vindelov, 1977). Fluorescence was quantified with a 488-nm argon laser FACStar flow cytometer (Becton Dickinson, Sunnyvale, CA, USA). A total of $2.0 \times 10^4$ nuclei were analysed for each sample, with a flow rate of approximately 150 nuclei per second. DNA aneuploidy was defined as a distinct peak separated from the diploid peak of the same tumour. The DNA index was determined as the ratio of the aneuploid mean channel number divided by the diploid $G_0/G_1$ mean channel number.

### Immunohistochemistry of Ki 67

Immunostaining of Ki 67 was performed using monoclonal antibody (NCL-Ki67-MM1; Novocastra Laboratories, Newcastle, UK) according to the avidin–biotin immunoperoxidase method. The sections were pretreated by microwaving them at 700 W for 5 min to retrieve the Ki 67 antigen then counterstained with 0.5% methyl green solution. The positive ratio of immunostaining for Ki 67 was evaluated using the Quantitative Estrogen/Progesteron Analysis software in the CAS 200 Image Analyser. The positive rate of immunostaining (PR) was expressed as the mean percentage of the tumour cells exhibiting positive staining in the total number measured in at least 20 different fields.

### Statistics

The Kruskal–Wallis test was used to examine the relationship between histological grade, tumour stage, venous involvement, DNA ploidy and chromosomal aberration of chromosomes 3, 7, 9 and 17 detected by FISH. The correlation of chromosomal aberration with Ki 67 expression was analysed statistically using a Pearson's correlation coefficient. A level of $P < 0.05$ was regarded as statistically significant.

### RESULTS

### Patients (Tables 1 and 2)

In normal controls, the percentage (mean ± s.d.) of nuclei with more than three signals for chromosomes 3, 7, 9 and 17 were 4.7 ± 0.8%, 4.1 ± 1.3%, 3.0 ± 1.8% and 2.7 ± 1.8% respectively. The percentage (mean ± s.d.) of nuclei with one signal for chromosomes

© Cancer Research Campaign 1998

3, 7, 9 and 17 were 3.9 ± 2.3%, 19.4 ± 20.5%, 16.1 ± 5.6% and 24.2 ± 7.3% respectively. The clinical parameters, DNA ploidy and Ki 67 expression of 21 RCCs are shown in Table 1. The positive rate of Ki 67 expression ranged from 0.62% to 34.6% (mean 8.9). Table 2 contains mean percentage of signal number for chromosomes 3, 7, 9 and 17 detected by FISH. Mean percentages of loss of chromosome 3 were significantly higher than those of chromosomes 7, 9 and 17 ($P < 0.001$, $P = 0.03$ and $P < 0.001$ respectively).

### Relationship of chromosomal aberrations detected by FISH with clinical parameters and DNA ploidy status (Tables 3 and 4)

The incidence of hyperdiploid aberration of chromosome 9 was significantly correlated with histological grade ($P = 0.04$) (Table 3). Hyperdiploid aberration of chromosomes 3 and 17 was seen more frequently in RCCs with advanced stage than those with low stage ($P = 0.04$, $P = 0.04$ respectively). The incidence of disomy of chromosome 7 was significantly lower in tumours exhibiting venous involvement than those not exhibiting venous involvement ($P = 0.04$). The remaining chromosomal aberrations were not interrelated with venous involvement. The incidence of hyperdiploid aberration of chromosomes 3, 7 and 17 was significantly lower in tumours with DNA diploidy than in those with DNA aneuploidy ($P = 0.02$, $P = 0.02$, $P = 0.002$ respectively). However, in chromosome 9 there was no correlation of chromosomal aberration with DNA ploidy.

### Relationship of chromosomal aberrations detected by FISH with Ki 67 expression (Table 4)

Ki 67 expression was closely related with hyperdiploid aberration of chromosomes 7 and 17 ($P = 0.01$, $P = 0.005$, $r = 0.58$, $r = 0.54$ respectively) and bore a reciprocal relation to disomy of chromosome 7 and monosomy of chromosome 17 ($P = 0.03$, $P = 0.04$, $r = -0.48$, $r = -0.46$ respectively).

### DISCUSSION

Recently, cytogenetic studies have revealed aberration of chromosome 7 to be a common event in RCC (Presti et al, 1991; Meloni et al, 1992). Beck et al (1995) reported that gain of chromosome 7 was found in 61% of RCC patients with DNA aneuploidy using FISH. However, the current results documenting a positive correlation between hyperdiploid aberration of chromosome 7 and DNA aneuploidy, as well as the correlation between aberration for chromosome 7 and venous involvement, appeared to suggest that gain and loss of chromosome 7 was significantly associated with a progress of the tumour invasion into vessels. Moreover, aberration of chromosome 7 was correlated with Ki 67 expression. Therefore, aberration of chromosome 7 appears to result from the process of gaining the metastatic potential as well as invading potential, which is a complicated phenomenon involving tumour cell proliferation.

In conventional RCC, the loss of chromosome 3p has been reported to be a frequent event (Meloni et al, 1992; Kovacs et al, 1989). In this study, loss of chromosome 3 was frequently observed and the mean percentage in loss of chromosome 3 was significantly higher than that observed in chromosomes 7, 9 and 17, which seems to be in agreement with the previous report described by Wu et al (1996), in which the loss of chromosome 3p was found in 90% of patients with RCC using the FISH technique. Therefore, the loss of chromosome 3 might represent a biological characteristic of RCC. However, the incidence of loss of chromosome 3 had no correlation with clinical parameters such as Ki 67 expression in this study. These findings suggested that the effect of loss of chromosome 3 on tumour development of RCC might be a rather early event. On the other hand, although there have been few reports regarding gain of chromosome 3 in RCC, our study demonstrated that gain of chromosome 3 was significantly correlated with advanced stage as well as DNA aneuploidy. Therefore, switching off the balance between gain and loss of chromosome 3 appeared to be significantly attributed to tumour development in RCC.

It has been reported that gain of chromosome 17 is a frequent cytogenic event in papillary RCC (Kovacs, 1993; Lager et al, 1995). Gain of chromosome 17 was significantly correlated with tumour stage. In addition, a significant correlation was noted between monosomy or hyperdiploid aberration of chromosomes 17 and Ki 67 expression in our study. p53 has been implicated as one of the tumour-suppressor genes and is located on chromosome 17p (Finlay et al, 1989). Mutation of the p53 gene frequently occurs in relation to neoplastic transformation is one of the steps involving malignant transformation and represents a malignant proliferation (Hollstein et al, 1991). Keeping this fact in mind, it might be quite reasonable that chromosomal gain or loss caused by inactivation of those genes is correlated with Ki 67 expression. Considering that the incidence of chromosome 17 abnormalities was increased along with tumour proliferation as evaluated by Ki 67 expression, our results seem to indicate that the aberration of chromosome 17 is a useful biological parameter for predicting disease progression.

In bladder tumours, as far as chromosome 9 was concerned, Yokogi et al (1996) reported that loss of chromosome 9 was frequently observed. In this study, a significant correlation was noted between hyperdiploid aberration of chromosome 9 and nuclear grade. Therefore, gain of chromosome 9, but not loss of chromosome 9, might be associated with tumour malignant behaviour. Further study was required to elucidate the relationship between hyperdiploid aberration of chromosome 9 and tumour progression in RCC.

The significance of tumour stage as a prognostic indicator for RCC is recognized (Nurmi, 1984), whereas it is controversial for a grading system to be reliable regarding prognostic relevance because of the interobserver variation (Fuhrman et al, 1982; Bretheau et al, 1995; Lanigan, 1995). However, our data demonstrated that specific chromosomal aberration detected by FISH was correlated with tumour stage and nuclear grade. These results might suggest that specific chromosomal aberrations are possible prognostic factors for RCC.

In summary, the preliminary results of our study revealed that the chromosomal aberrations as assessed by the FISH technique appears to provide a new clinical insight into determination of the biological potential of RCCs, considering that (1) chromosomal aberrations as detected by FISH were interrelated with clinicopathological features and (2) some were associated with Ki 67 expression. In particular, it is suggested that gain of a chromosome might be clinically relevant for predicting the clinical outcome of patients with RCC. Multivariate survival analysis including a larger series of RCCs would clarify the prognostic significance of chromosomal aberration as detected by FISH.

*© Cancer Research Campaign 1998*

### REFERENCES

Arner O, Blanck C and von Schreeb T (1965) Renal adenocarcinoma: morphology, grading of malignancy, prognosis. A study of 197 cases. *Acta Chir Scand* **346** (Suppl.): 1–51

Beck JML, Hopman AHN, Feitz WFJ, Schalken J, Schaafsma HE, van de Kaa CA, Ramaekers FCS, Hanselaar AGJM and de Wilde PCM (1995) Numerical aberrations of chromosomes 1 and 7 in renal cell carcinomas as detected by interphase cytogenetics. *J Pathol* **176**: 123–135

Bretheau D, Lechevallier E, de Fromont M, Sault MC, Rampal M and Coulange C (1995) Prognostic value of nuclear grade of renal cell carcinoma. *Cancer* **76**: 2543–2549

Cher ML, Ito T, Weidner N, Carroll PR and Jensen RH (1995) Mapping of regions of physical deletion on chromosome 16q in prostate cancer cells by fluorescence *in situ* hybridization (FISH). *J Urol* **153**: 249–254

Drach J, Schuster J, Nowotny H, Angerler J, Rosenthal F, Fiegl M, Rothermundt C, Gsur A, Jager U, Heinz R, Lechner K, Ludwig H and Huber H (1995) Multiple myeloma: high incidence of chromosomal aneuploidy as detected by interphase fluorescence *in situ* hybridization. *Cancer Res* **55**: 3854–3859

Finlay CA, Hinds PW and Levine AJ (1989) The p53 proto-oncogene can act as a suppressor of transformation. *Cell* **57**: 1083–1093

Fuhrman SA, Lasky LC and Limas C (1982) Prognostic significance of morphologic parameters in renal cell carcinoma. *Am J Surg Pathol* **6**: 655–663

Gomyo Y, Andachi H, Nagao K, Ikeguchi M and Ito H (1995) Interphase cytogenetics of gastric carcinoma: fluorescence *in situ* hybridization (FISH) applied to cells obtained from formalin-fixed paraffin-embedded tissues. *Pathol Int* **45**: 227–232

Hermanek P, Scheibe O, Spiessl B and Wagner G (1987) TNM Klassification maligner Tumoren, UICC. Springer: New York.

Hollstein M, Sidransky D, Vogelstein B and Harris CC (1991) p53 mutations in human cancers. *Science* **253**: 49–53

Hopman AHN, Poddighe PJ, Smeets AWGB, Moesker O, Beck JLM, Vooijs GP and Ramaekers FCS (1989) Detection of numerical chromosome aberrations in bladder cancer by *in situ* hybridization. *Am J Pathol* **135**: 1105–1117

Hopman AHN, Moesker O, Smeets AWGB, Pauwels RPE, Vooijs GP and Ramaekers FCS (1991) Numerical chromosome 1, 7, 9, and 11 aberrations in bladder cancer detected by *in situ* hybridization. *Cancer Res* **51**: 644–651

Kovacs G (1993) Molecular differential pathology of renal cell tumours. *Histopathology* **22**: 1–8

Kovacs G, Wilkens L, Papp T and De Riese W (1989) Differentiation between papillary and nonpapillary renal cell carcinomas by DNA analysis. *J Natl Cancer Inst* **81**: 527–530

Lager DJ, Huston BJ, Timmerman TG and Bonsib SM (1995) Papillary renal tumours. *Cancer* **76**: 669–673

Lanigan D (1995) Prognostic factors in renal cell carcinoma. *Br J Urol* **75**: 565–571

Meloni AM, Bridge J and Sandberg AA (1992) Reviews on chromosome studies in urological tumors. 1. Renal tumors. *J Urol* **148**: 253–265

Nurmi MJ (1984) Prognostic factors in renal carcinoma: an evaluation of operative findings. *Br J Urol* **56**: 270–275

Papadopoulos I, Weichert-Jacobsen K, Nurnberg N and Sprenger E (1995) Quantitative DNA analysis in renal cell carcinoma. Comparison of flow and image cytometry. *Anal Quant Cytol Histol* **17**: 272–275

Pinkel D, Straume T and Gray JW (1986) Cytogenetic analysis using quantitative, high-sensitivity, fluorescence hybridization. *Proc Natl Acad Sci* **83**: 2934–2938

Presti Jr JC, Rao PH, Chen Q, Reuter VE, Li FP, Fair WR and Jhanwar SC (1991) Histopathological, cytogenetic, and molecular characterization of renal cortical tumors. *Cancer Res* **51**: 1544–1552

Schutte B, Reynders MM, Bosman FT and Blijham GH (1985) Flow cytometric determination of DNA ploidy level in nuclei isolated from paraffin-embedded tissue. *Cytometry* **6**: 26–30

Shackney SE, Singh SG, Yakulis R, Smith CA, Pollice AA, Petruolo S, Waggoner A and Hartsock RJ (1995) Aneuploidy in breast cancer: a fluorescence *in situ* hybridization study. *Cytometry* **22**: 282–291

Syrjanen K and Hjelt L (1978) Grading of human renal adenocarcinoma. *Scand J Urol Nephrol* **12**: 49–55

Veloso JD, Solis OG, Barada JH, Fisher HA and Ross JS (1992) DNA ploidy of oncocytic-granular renal cell carcinomas and renal oncocytomas by image analysis. *Arch Pathol Lab Med* **116**: 154–158

Vindelov LL (1977) Flow microfluorometric analysis of nuclear DNA in cells from solid tumors and cell suspensions. A new method for rapid isolation and straining of nuclei. *Virchows Arch Cell Pathol* **24**: 227

Visakorpi T, Hyytinen E, Kallioniemi A, Isola J and Kallioniemi O (1994) Sensitive detection of chromosome copy number aberrations in prostate cancer by fluorescence *in situ* hybridization. *Am J Pathol* **145**: 624–630

Wu S, Hafez GR, Xing W, Newton M, Chen XR and Messing E (1996) The correlation between the loss of chromosome 14q with histological tumor grade, pathological stage, and outcome of patients with nonpapillary renal cell carcinoma. *Cancer* **77**: 1154–1160

Yokogi H, Wada Y, Moriyama-Gonda N, Igawa M and Ishibe T (1996) Genomic heterogeneity in bladder cancer as detected by fluorescence *in situ* hybridization. *Br J Urol* **78**: 699–703

© Cancer Research Campaign 1998